# A *DAAM1* 3′-UTR SNP mutation regulates breast cancer metastasis through affecting miR-208a-5p-DAAM1-RhoA axis

**DOI:** 10.1186/s12935-019-0747-8

**Published:** 2019-03-11

**Authors:** Jie Mei, Ting Yan, Yifu Huang, Tiansong Xia, Fei Chang, Shuning Shen, Leiyu Hao, Yin Chen, Zhongyuan Wang, Xiaozheng Jiang, Bujie Xu, Yichao Zhu

**Affiliations:** 1 Department of Physiology, Nanjing Medical University, 101 Longmian Road, Nanjing, 211166 China; 2 Safety Assessment and Research Center for Drug, Pesticide and Veterinary Drug of Jiangsu Province, Nanjing Medical University, Nanjing, 211166 China; 3 Department of Prevention and Healthcare, Affiliated Jiangyin Hospital of Southeast University Medical College, Jiangyin, 214400 China; 4 Breast Disease Center, The First Affiliated Hospital with Nanjing Medical University, Nanjing, 210036 China; 5 State Key Laboratory of Reproductive Medicine, Nanjing Medical University, Nanjing, 211166 China

**Keywords:** DAAM1, 3′-UTR, rs79036859, miR-208a-5p, Metastasis

## Abstract

**Background:**

Dishevelled-associated activator of morphogenesis 1 (DAAM1) is a member of microfilament-related formins and mediates cell motility in breast cancer (BrCa). However, the genetic mutation status of *DAAM1* mRNA and its correlation with pathological characteristics are still unclearly. Methods: A patient cohort and BrCa cells were recruited to demonstrate the role of functional SNP in microRNA-208a-5p binding site of *DAAM1* 3′-UTR and underlying mechanism in BrCa metastasis.

**Methods:**

A patient cohort and BrCa cells were recruited to demonstrate the role of functional SNP in microRNA-208a-5p binding site of *DAAM1* 3′-UTR and underlying mechanism in BrCa metastasis.

**Results:**

The expression and activation of DAAM1 increased markedly in lymphnode metastatic tissues. A genetic variant (rs79036859 A/G) was validated in the miR-208a-5p binding site of *DAAM1* 3′-UTR. The G genotype (AG/GG) was a risk genotype for the metastasis of BrCa by reducing binding affinity of miR-208a-5p for the *DAAM1* 3′-UTR. Furthermore, the miR-208a-5p expression level was significantly suppressed in lymphnode metastatic tissues compared with that in non-lymphnode metastatic tissues. Overexpression of miR-208a-5p inhibited DAAM1/RhoA signaling pathway, thereby leading to the decrease of the migratory ability.

**Conclusion:**

Overall, the rs79036859 G variant of *DAAM1* 3′-UTR was identified as a relevant role in BrCa metastasis via the diversity of miR-208a-5p binding affinity.

**Electronic supplementary material:**

The online version of this article (10.1186/s12935-019-0747-8) contains supplementary material, which is available to authorized users.

## Background

Dishevelled-associated activator of morphogenesis 1 (DAAM1) is a member of microfilament-related formins and is involved in cell motility through mediating Wnt signaling pathway [1–3]. In the cytoplasm, DAAM1 exists in an autoinhibited state by intramolecular interaction between its N-terminal GBD and C-terminal DAD domains. When Dishevelled 2 binds to DAAM1, leading to disrupting the interaction between the GBD and DAD that mediates DAAM1 auto-inhibition, DAAM1 will exposure FH1 and FH2 domains to polymerize straight actin filaments [2, 4]. DAAM1 is essential for Wnt-11/Frizzled-induced activation of RhoA and *Xenopus* gastrulation [2]. DAAM1 directly collaborates to fascin in actin filaments and thus controls the formation of filopodia [5]. Our previous studies find that active DAAM1 is involved in Wnt5a/Dishevelled 2 and Collagen/Integrin αvβ3 signaling pathways, resulting in the elevation of the migratory and haptotatic ability of breast cancer (BrCa) cells [3, 6]. However, the genetic mutation status of DAAM1 mRNA and its correlation with pathological characteristics are still unknown in BrCa patients.

Single nucleotide polymorphisms (SNPs) located in untranslated region (UTR) have been reported to be associated with dysregulation of genes expression. A recent global transcriptional network study identifies mutations at somatic expression quantitative trait locus (eQTL) located 5′-UTR of *DAAM1*, directly regulating the expression of *DAAM1* mRNA [7]. Nevertheless, noncoding mutations in the 3′-UTR of *DAAM1* has not been reported. An increasing evidence revealed that functional 3′-UTR SNPs are participated in the progression of multiple cancers [8–11]. Most 3′-UTR of mRNAs function as the target sequences of microRNAs (miRNAs) by base pairing, thereby leading to the degradation of mRNAs and decrease of their translation [12, 13]. SNPs in the miRNA binding sites in the 3′-UTRs of target genes represent their differential binding affinities for corresponding miRNAs [14–16].

Here, we demonstrate that DAAM1 is highly expressed in lymphnode metastatic BrCa tissues. We also elucidate the functional role of SNP rs79036859 in the miR-208a-5p binding site of *DAAM1* 3′-UTR and its involvement in BrCa metastasis. Overall these data identify miR-208a-5p/DAAM1 axis as a potential therapeutic target in limiting BrCa metastasis and reducing death from this disease.

## Methods

### Clinical information

157 BrCa patients were recruited by the First Affiliated Hospital with Nanjing Medical University and Affiliated Cancer Hospital to Nanjing Medical University (NJMU) from 2015 to 2018. All cases had been diagnosed with BrCa by a pathologist on the basis of hematoxylin–eosin (HE) staining. Relevant clinicopathological characteristics record for each case were collected by review of patients’ medical files. Ethical approval for the study was obtained from the Clinical Research Ethics Committee, NJMU. Pathologic staging was determined by a pathologist based on AJCC Cancer Staging Manual 8th classification criteria. All the participants voluntarily joined this study with informed contents.

### Immunohistochemistry (IHC)

A total of 46 BrCa sections were deparaffinised at 55 °C for 30 min. The sections were then washed with xylene for three 5-min. The sections were rehydrated by successive washes in 100, 90 and 70% graded ethanol. Hydrogen peroxidase (0.3%, ZSGB-Bio, Beijing, China) was used to block endogenous peroxidase activity for 20 min. The primary anti-DAAM1 (1:100 dilution, Cat. 14876-1-AP, ProteinTech, Wuhan, China) antibody and DAB and hematoxylin counterstain (ZSGB-Bio) were used to visualize its expression. The percentage of positively stained cells was scored as 0–4: 0 (< 5%), 1 (6–25%), 2 (26–50%), 3 (51–75%) and 4 (> 75%). The staining intensity was scored as 0–3: 0 (negative), 1 (weak), 2 (moderate), and 3 (strong). The immunoreactivity score (IRS) equals to the percentages of positive cells multiplied with staining intensity. Immunostained sections were scanned by a microscope (Olympus Corporation, Tokyo, Japan).

### Selection of SNPs and TaqMan genotyping

A next-generation sequencing of metastatic BrCa tissues (data not published) revealed some SNPs, including rs79036859 and rs45476291, locating in the 3′-UTR of *DAAM1*. After a review of dbSNP database (https://www.ncbi.nlm.nih.gov/snp) and PolymiRTS Database 3.0 (http://compbio.uthsc.edu/miRSNP), we selected the candidate SNP (rs79036859) suggested as a transcriptional regulation factor for the 3′-UTR of *DAAM1*. SNP genotyping was conducted by allelic discrimination using the TaqMan SNP Genotyping Assays according to the manufacturer’s instructions (Applied Biosystems). Specific primers (TATCTCCTGAAAGAGATAAGA, GTTTTTCCAACAACTCCAGT) and FAM/VIC-labeled TaqMan probes (FAM-labeled CAAACAAACAAAAAAAGCTTGCAAAATATTTT, VIC-labeled CAAACAAACAAAAAAGGCTTGCAAAATATTTT) were designed and supplied by Synbio Technologies (Soochow, China). The PCR conditions were as follows: initiation at 98 °C for 10 min, followed by 40 cycles of denaturation at 95 °C for 30 s and annealing/extension at 60 °C for 60 s. PCR application was undergoing in a StepOnePlus Real-Time PCR System (Applied Biosystems).

### Cell culture and transfection

MCF-10A, MDA-MB-231, MCF-7, and COS-7 cell lines were purchased from the Cell Bank of Chinese Academy of Sciences (Shanghai, China). MCF-7, MDA-MB-231, and COS-7 cells were maintained in Dulbecco’s modified Eagle’s medium (DMEM, high glucose) (Hyclone, Thermo Scientific, Waltham, MA) supplemented with 10% (v/v) fetal bovine serum (FBS) (Hyclone) at 37 °C with 5% CO_2_. MCF-10A cells were cultured in DMEM/F12 media supplemented with 5% (v/v) horse serum, 20 ng/mL human EGF, 10 μg/mL insulin, 0.5 μg/mL hydrocortisone, penicillin, streptomycin and 100 ng/mL cholera toxin (Sigma-Aldrich, St. Louis, MO).

For subsequent assays, cells were transfected with miR-208a-5p mimic, miR-208a-5p inhibitor (an anti-sense of miR-208a-5p), or miRNA mimic control, which is synthesized in RiboBio Co., Ltd. (Guangzhou, China), using Lipofectamine 2000 Reagent (Invitrogen, Carlsbad, CA) according to the manufacturer’s instructions.

### Western blotting analysis and pulldown assays

Cells were placed in 35-mm dishes (6 × 10^5^ cells/dish) and transfected with synthesized miR-208a-5p mimic, miR-208a-5p inhibitor (anti-sense of miR-208a-5p), or miRNA mimic control. 72 h after transfection, all cells were harvested the proteins with lysis buffer. SDS–polyacrylamide gel electrophoresis and Western blotting analysis were performed as standard protocols. The primary antibodies for DAAM1 (1:1000 dilution, Cat. 14876-1-AP, ProteinTech), RhoA (1:1000 dilution, Cat. 10749-1-AP, ProteinTec), β-actin (1:5000 dilution, Cat. 60008-1-Ig, ProteinTec) were used. DAAM1 protein levels were normalized to β-actin for each sample.

To detect the active level of DAAM1, we employed GST-RhoA beads as bait. The activate level of DAAM1 was detected by the Pulldown assays and subsequently immunoblotted with anti-DAAM1 antibody (Cat. 14876-1-AP, ProteinTech) [6]. SDS-PAGE and Western blotting were performed using the above methods.

### Dual-luciferase activity assay

The 3′-UTR of *DAAM1* which contained the putative target site of miR-208a-5p was synthesized by Integrated Biotech Solutions Co., Ltd (Shanghai, China) and ligated into pGL3 construct (Promega, Madison, WI). The constructs pGL3-DAAM1-3′-UTR-WT or pGL3-DAAM1-3′-UTR-mutant (200 ng) and pRL-TK (80 ng, Promega) were co-transfected with 60 pmol miR-208a-5p mimic or miRNA mimic control by Lipofectamine 2000 (Invitrogen). Twenty-four hours after transfection, Dual-Luciferase Reporter Assay System (Promega) was performed to measure luciferase activity.

### Boyden chamber assays

Migratory ability of cancer cells was tested in a modified Boyden chamber (Cat. 3422, Costar, Corning, NY). The detail protocol was described as previously [6].

### Immunofluorescence and actin cytoskeleton staining

Cells were placed on glass cover-slides and subjected to actin cytoskeleton staining. The detail protocol was described as previously [17]. The images were captured by a laser scanning confocal microscope (Zeiss LSM710, Oberkochen, Germany).

### Quantitative real-time PCR

MiRNA of BrCa cells and tissues were extracted by using mirVana™ miRNA isolation kit (Ambion, Austin, TX). The primers for miRNA reverse transcription were synthesized in RiboBio Co., Ltd. (Guangzhou, China) called Bulge-Loop™ miRNA qRT-PCR Primer Set as previously described [17]. Primers used for *DAAM1* amplification were *GAPDH*: 5′-TGAACGGGAAGCTCACTGG-3′ (sense) and 5′-TCCACCACCCTGTTGCTGTA-3′ (antisense); *DAAM1*: 5′-AAATTGAAACGGAATCGCAAAC-3′ (sense) and 5′-GCAAGGCAGTGTAATGAAACG-3′ (antisense). SYBR Green (SYBR^®^ Premix Ex Taq™ II, TaKaRa, Dalian, China) was used to label the amplified genes. The 2^−ΔΔCt^ method was used for miR-208a-5p or *DAAM1* expression analysis.

### RhoA GTPase activation assays (G-LISA small GTPase activation assays)

Total protein lysates extracted from BrCa cells were turned to measure RhoA activity by using RhoA GTPase activation assays (Cat. BK121, Cytoskeleton Inc., Denver, CO). RhoA activation was described as previously [6].

### Kaplan–Meier plotter analysis

*DAAM1* (Affy ID: 216060_s_at) mRNA expression data and survival information (progression free survival, overall survival, post progression survival and distant metastasis free survival) were collected from Kaplan–Meier plotter (http://kmplot.com). Then, the prognostic significance of *DAAM1* mRNA was analyzed in BrCa. Kaplan–Meier survival plots were generated with survival curves compared by log-rank test.

### Statistical analysis

All statistical analyses were calculated using SPSS 25.0 software (Chicago, IL). Most of the data were analyzed by Student’s *t*-test or one-way ANOVA followed by Dunnett’s multiple posthoc tests. All data are presented as means ± SDs of five independent experiments if not noted. The associations between *DAAM1* mRNA expression levels, genotypes distribution of *DAAM1* SNP, along with miR-208a-5p expression status and clinicopathological characteristics were performed using Pearson’s Chi squared test. The correlations between *DAAM1* mRNA expression and miR-208a-5p expression or between *DAAM1* mRNA level and protein level were assessed by Spearman’s correlation analysis.

## Results

### DAAM1 expression positively correlates with lymphnode metastasis and associates with prognosis in BrCa

**Fig. 1:**
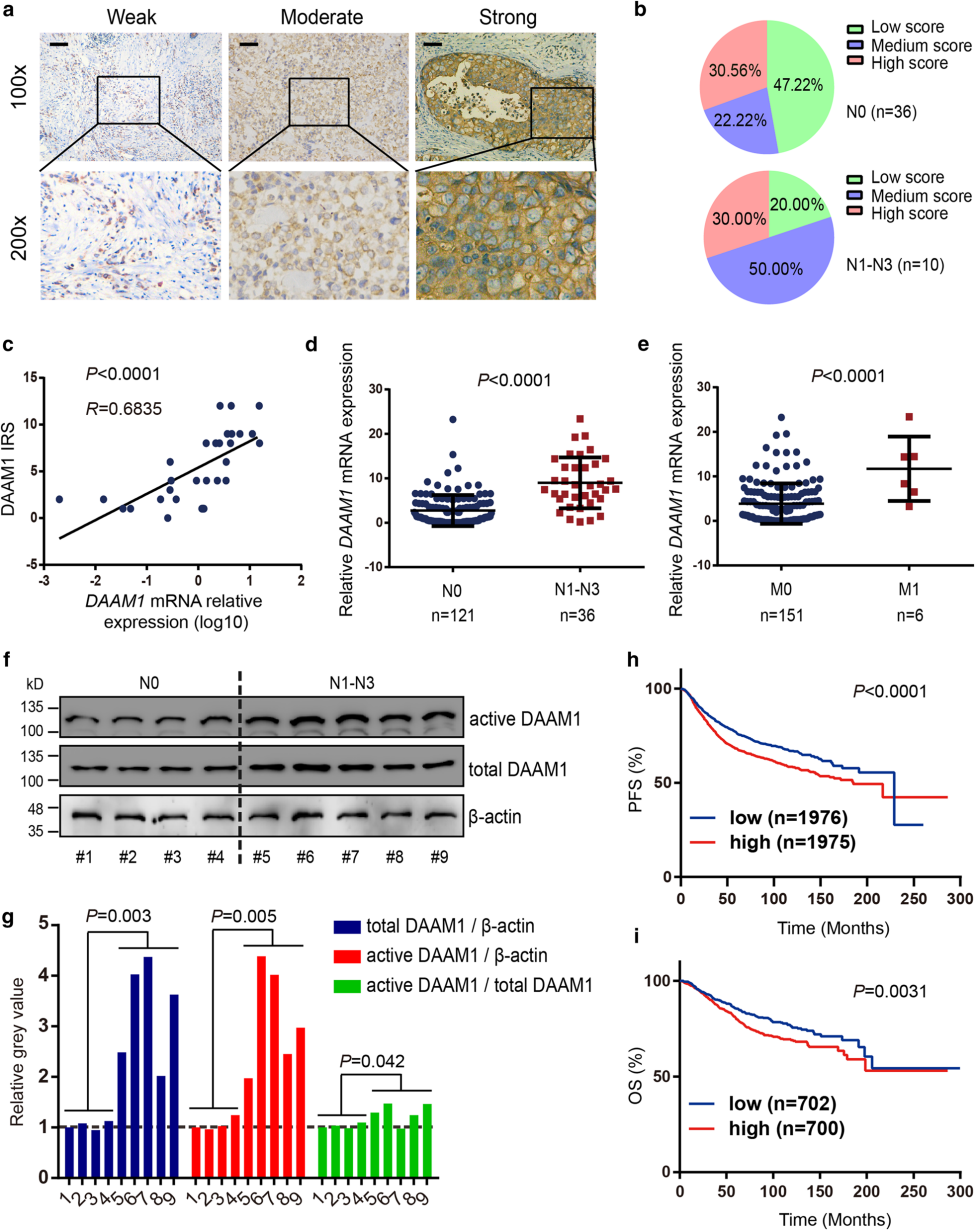
The expression of DAAM1 are relative with the high rate of lymphnode metastasis and associated with prognosis in BrCa. **a** Representative microphotographs revealing low, medium, and high DAAM1 expression using immunohistochemical (IHC) staining. Brown, DAAM1. Blue, haematoxylin. Bar = 100 μm. Objective lens, magnification, ×10; numerical aperture, 0.75. **b** DAAM1 protein expression intensity proportion based on lymphnode metastasis. N0, without lymphnode metastasis. N1–N3, with lymphnode metastasis. **c** Correlation between the expression levels of DAAM1 protein and *DAAM1* mRNA in BrCa tissues. **d**, **e** The expression of *DAAM1* mRNA according to lymphnode metastasis and distance metastasis. M0, without distance metastasis. M1, with distance metastasis. **f** The lysates of tumor tissues were assayed for the expression levels of total and active DAAM1 by Western blotting and pulldown assay using a GST-RhoA as a bait, respectively. β-actin was used as the loading control. **g** The expression ratios of total DAAM1/β-actin, active DAAM1/β-actin, and active DAAM1/total DAAM1. **h**, **i** Kaplan–Meier analysis showed survival curves of progression free survival (PFS) and overall survival (OS) and of patients with low *DAAM1* mRNA expression *vs* high expression in BrCa

Our previous studies reported that DAAM1 regulates the re-organization of microfilaments for oriented the migration and haptotaxis of BrCa cells [3, 6]. However, it is still limited evidence demonstrating that DAAM1 is correlated with tumor metastasis in BrCa patients. To investigate whether DAAM1 acts as a metastatic promoter in BrCa, the immunohistochemistry (IHC) were performed to test the DAAM1 expression in 46 BrCa cases. The sections highly and moderately expressed DAAM1 accounted for a majority of lymphnode metastatic cases (Fig. 1a, b).

Because of limited amount of IHC sections, we further examined the transcriptional level of *DAAM1* in 157 BrCa samples. We firstly determined that *DAAM1* mRNA expression was positively correlated with DAAM1 protein expression (Fig. 1c). When we compared *DAAM1* mRNA expression in lymphnode metastatic tumor tissues and non-lymphnode metastatic tumor tissues along with distance metastatic tumor tissues and non-distance metastatic tumor tissues, the expression of *DAAM1* mRNA was significantly increased in metastatic tissues (Fig. 1d, e, Table 1). Furthermore, we tested DAAM1 expression and DAAM1 activity in fresh BrCa tissues (Fig. 1f). We found that both the expression and the activation of DAAM1 in lymphnode metastatic tumor tissues were significantly higher than these in non-lymphnode metastatic tumor tissues (Fig. 1f, g).

**Table 1:** Associations between *DAAM1* mRNA expression and clinicopathological characteristics in breast cancer

Characteristics	n	DAAM1^a^		OR (95% Cl)	*P* value^b^
		Low	High		
Tumor size (cm)					
≤ 2	61	35	26	1.82 (0.94–3.53)	0.075
> 2	87	50	50		
Unknown	9				
Lymph node metastasis					
N0	121	73	48	9.43 (3.43–25.95)	< *0.001*
N1–N3	36	5	31		
Distant metastasis					
M0	151	78	73	–	*0.039*
M1	6	0	6		
ER status					
Negative	51	23	28	0.76 (0.39–1.49)	0.426
Positive	106	55	51		
PR status					
Negative	67	31	37	0.77 (0.41–1.45)	0.419
Positive	89	47	42		
Unknown	1				
Her-2 status					
Negative	94	45	49	0.89 (0.47–1.69)	0.720
Positive	61	31	30		
Unknown	2				
P53 status					
Negative	105	51	54	0.99 (0.48–2.04)	0.982
Positive	41	20	21		
Unknown	11				
Ki67 status					
Negative	33	19	14	1.53 (0.70–3.33)	0.285
Positive	119	56	63		
Unknown	5				

Italic values indicate significance of* P* value (*P* < 0.05)

^a^The *DAAM1* mRNA expression levels were divided at a cutoff point of 50%

^b^*P* value for χ^2^ test

Moreover, both progression free survival (PFS) and overall survival (OS) rate of BrCa patients with high expression of *DAAM1* mRNA was significantly worse than that of patients with low *DAAM1* expression according to survival information from Kaplan–Meier plotter (Fig. 1h, i). Overall, the clinical data reveals that DAAM1 functions as a metastatic promoter and associates with prognosis in BrCa.

### G genotype of rs79036859 enhances the risk of metastasis in BrCa

**Fig. 2:**
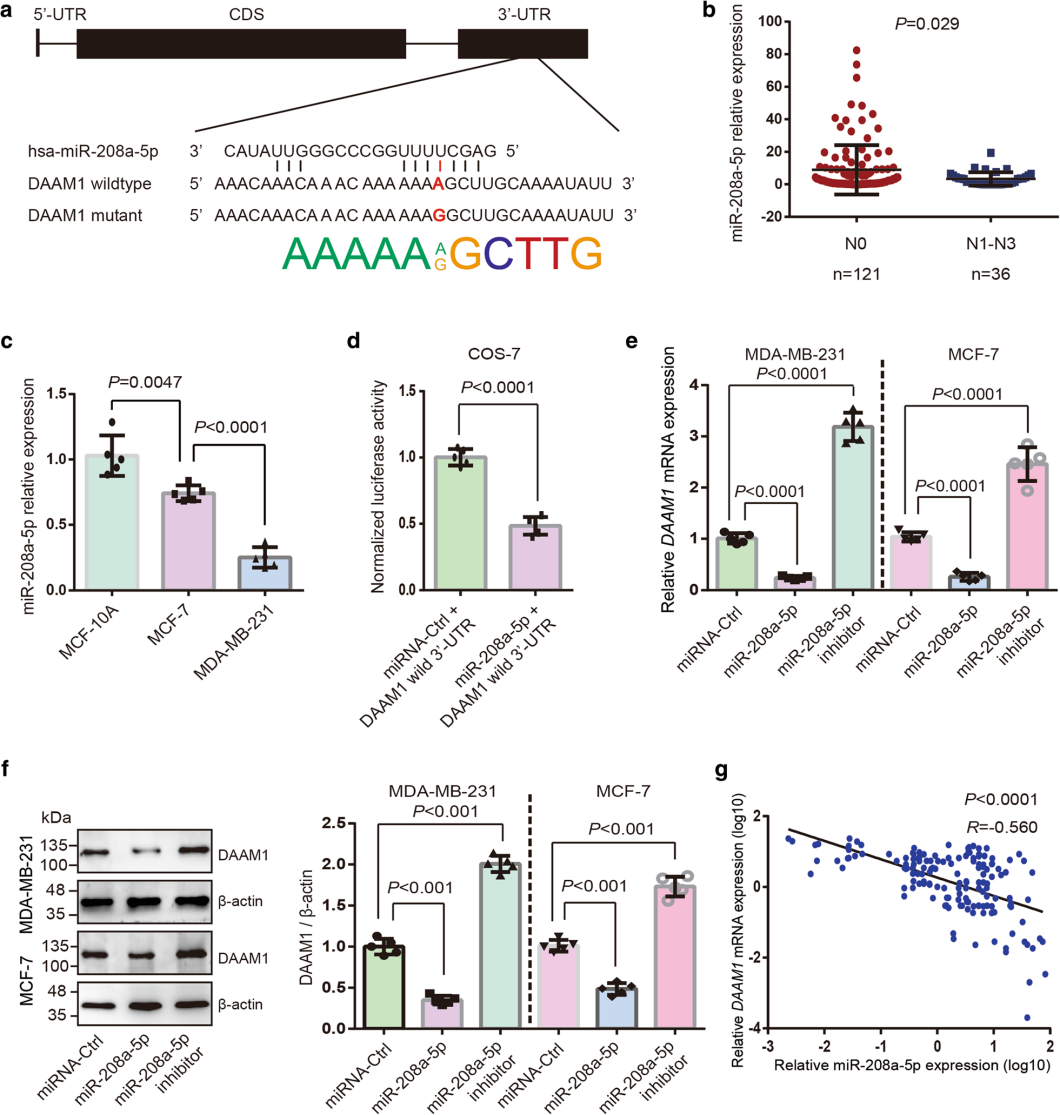
MiR-208a-5p is a metastatic suppressor and downregulates DAAM1. **a** *DAAM1* gene structure and A/G polymorphism in the *DAAM1* 3′-UTR at the miR-208a-5p binding sites. **b** The miR-208a-5p expression in lymphnode metastatic BrCa tissues and non-metastatic BrCa tissues. N0, without lymphnode metastasis. N1–N3, with lymphnode metastasis. **c** MiR-208a-5p exhibited the lower expression level in MCF-7 and MDA-MB-231 BrCa cells than that in MCF-10A mammary epithelial cells. **d** The luciferase activity in COS-7 cells transfected with wild type *DAAM1* 3′-UTR (DAAM1 wild 3′-UTR) and miR-208a-5p or miRNA-Ctrl. **e**, **f** The downregulated DAAM1 both in mRNA and protein levels after miR-208a-5p overexpression, and the upregulated DAAM1 protein expression after transfection of miR-208a-5p inhibitor (anti-sense of miR-208a-5p) in MDA-MB-231 and MCF-7 BrCa cells. β-actin was used as the loading control. **g** Correlation between the expression levels of miR-208a-5p and *DAAM1* mRNA in BrCa tissues

In view of a fact that DAAM1 showed the increased expression and activation level in lymphnode metastatic tumor tissues, we speculated that the regulation of genomic and/or transcriptional level may be heterogeneous in individual BrCa tissues. After a review of dbSNP database and PolymiRTS Database 3.0 [18], SNP rs79036859 was predicted as the regulatory element for the post-transcription of *DAAM1* expression (Fig. 2a).

To further verify the potential effect of the *DAAM1* 3′-UTR SNP rs79036859 in BrCa, we performed a case-based method to assess the function of rs79036859 in tumor metastasis. A metastatic cases-control study including 157 BrCa cases (121 cases without lymphnode metastasis and 36 cases with lymphnode metastasis) was proceeded. BrCa patients with the G (AG/GG) allele genotypes had a prominently higher risk of metastasis compared with the AA genotype carriers (OR = 3.90, 95% CI 1.77–8.59; *P *< 0.001; Table 2). These findings demonstrate that G genotype in rs79036859 indicates a high risk of metastasis in BrCa.

**Table 2:** Clinicopathological characteristics, allelic, and genotypes distribution in breast cancer patients

Characteristics	n	rs79036859^a^		OR (95% Cl)	*P* value^b^
		AA	AG/GG		
Tumor size (cm)					
≤ 2	61	37	24	1.20 (0.61–2.33)	0.599
> 2	87	49	38		
Unknown	9				
Lymph node metastasis					
N0	121	70	41	3.90 (1.77–8.59)	< *0.001*
N1–N3	36	12	24		
Distant metastasis					
M0	151	90	61	2.95 (0.52–16.62)	0.200
M1	6	2	4		
ER status					
Negative	51	29	22	0.90 (0.46–1.77)	0.759
Positive	106	63	43		
PR status					
Negative	67	38	29	0.85 (0.45–1.62)	0.619
Positive	89	54	35		
Unknown	1				
Her-2 status					
Negative	94	51	43	0.67 (0.35–1.30)	0.233
Positive	61	39	22		
Unknown	2				
P53 status					
Negative	105	63	42	1.30 (0.63–2.68)	0.485
Positive	41	22	19		
Unknown	11				
Ki67 status					
Negative	33	22	11	1.55 (0.69–3.49)	0.285
Positive	119	67	52		
Unknown	5				

Italic values indicate significance of* P* value (*P* < 0.05)

^a^AA genotype was used as a reference

^b^*P* value for χ^2^ test

### SNP rs79036859 associated miR-208a-5p is downregulated in BrCa tissues and directly target *DAAM1*

Because of the SNP rs79036859 associated miR-208a-5p predicted as post-transcriptional regulatory factor of DAAM1 (Fig. 2a), we hypothesized that miR-208a-5p would be a metastatic suppressor by downregulating DAAM1. We examined miR-208a-5p expression in 36 metastatic BrCa tissues and 121 non-metastatic BrCa tissues using quantitative PCR. The miR-208a-5p expression level in lymphnode metastatic tumor tissues was significantly lower than that in non-lymphnode metastatic tumor tissues (Fig. 2b). The expression status of miR-208a-5p was not significantly associated with clinicopathological parameters of BrCa, including tumor size, lymph node metastasis, distant metastasis, ER, PR, Her2, P53, and Ki67 status by using χ^2^ test analysis (Additional file 1: Table S1). Furthermore, we examined miR-208a-5p expression in cell lines. MiR-208a-5p exhibited the lower expression level in MCF-7 and MDA-MB-231 BrCa cells than that in MCF-10A mammary epithelial cells (Fig. 2c).

To further verify the binding of miR-208a-5p and *DAAM1* 3′-UTR, we performed dual-luciferase activity assays. A notable decline of luciferase activity was shown in DAAM1 wild 3′-UTR and miR-208a-5p mimic overexpressed group, and disappeared in miRNA-control groups (Fig. 2d). Next, MDA-MB-231 and MCF-7 cells were transfected with FAM-labelled miR-208a-5p mimic, miR-208a-5p inhibitor (anti-sense of miR-208a-5p), or miRNA mimic control. The mRNA and protein level of DAAM1 was significantly decreased in miR-208a-5p-overexpressed in MCF-7 and MDA-MB-231 cells, but upregulated in miR-208a-5p inhibitor-overexpressed cancer cells (Fig. 2e, f). Besides, miR-208a-5p expression was inversely correlated with *DAAM1* expression in 157 clinical samples (Fig. 2g). These results indicated that miR-208a-5p functions as metastatic suppressor and directly targets *DAAM1* in BrCa.

### SNP rs79036859 A/G eliminates miR-208a-5p-mediated decrease of *DAAM1* expression

**Fig. 3:**
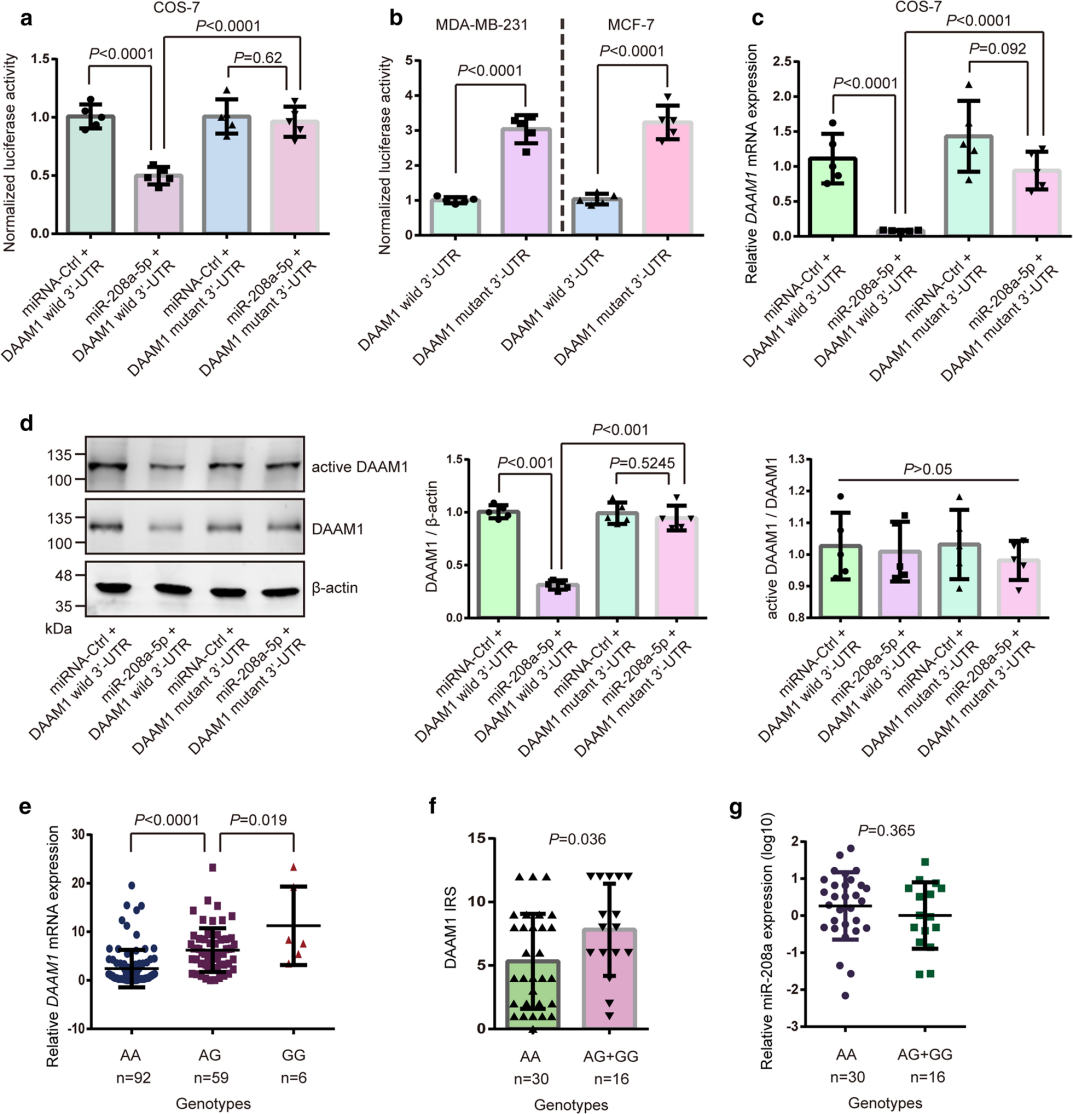
SNP rs79036859 A/G eliminates miR-208a-5p-mediated decrease of *DAAM1* expression. **a** The luciferase activity in COS-7 cells transfected with wild type *DAAM1* 3′-UTR (DAAM1 wild 3′-UTR) or the mutant of *DAAM1* 3′-UTR SNP rs79036859 A → G (DAAM1 mutant 3′-UTR) and miR-208a-5p. **b** The luciferase activity in both MDA-MB-231 and MCF-7 cells transfected with the A allele construct (wild type *DAAM1*) and the reporter bearing the G allele (mutant *DAAM1*). **c**, **d** The downregulated DAAM1 both in mRNA and protein levels COS-7 cells transfected with wild type *DAAM1* 3′-UTR (DAAM1 wild 3′-UTR) or the mutant of *DAAM1* 3′-UTR SNP rs79036859 A → G (DAAM1 mutant 3′-UTR) and miR-208a-5p. The level of active DAAM1 was measured and non-significant difference in ratio of active DAAM1/total DAAM1 were found in above group. β-actin was used as the loading control. **e** The expression levels of *DAAM1* mRNA in BrCa samples with AA, AG, and GG genotypes. **f** According to the DAAM1 expression using IHC staining, G (AG + GG) genotypes had a higher IRS level of DAAM1 than the wild type AA genotype. **g** The non-significant difference in miR-208a-5p expression levels were found in wild type AA genotype and mutant G (AG + GG) genotypes in 46 samples

Rs79036859 may regulate *DAAM1* expression via altering the binding affinity of miR-208a-5p for two *DAAM1* 3′-UTR genotypes. A notable decline of luciferase activity was shown in DAAM1 wild 3′-UTR and miR-208a-5p mimic transfected group, but vanished in DAAM1 mutant 3′-UTR groups (Fig. 3a). Moreover, DAAM1 wild 3′-UTR or DAAM1 mutant 3′-UTR transfected into MDA-MB-231 and MCF-7 cells. We found that the relative luciferase activity in DAAM1 mutant 3′-UTR-expressed cells was higher than that in DAAM1 wild 3′-UTR-expressed cells (Fig. 3b). Compared with the DAAM1 mutant 3′-UTR group, DAAM1 expression was suppressed both in mRNA and protein level in cells transfected with DAAM1 wild 3′-UTR and miR-208a-5p mimic (Fig. 3c, d). Besides, the level of active DAAM1 was also significantly downregulated in cells transfected with DAAM1 wild 3′-UTR and miR-208a-5p mimic, but non-significant difference in the ratio of active DAAM1/total DAAM1 were found in above group (Fig. 3d).

To assess whether rs79036859 A/G was related to DAAM1 expression in clinical samples, we detected *DAAM1* mRNA levels in cancer tissues using quantitative PCR in 157 cases and examined DAAM1 protein expression by IHC in 46 patients. The results showed that *DAAM1* mutant tumor tissues (AG/GG genotypes) expressed higher levels of DAAM1 mRNA and protein than *DAAM1* wildtype tumor tissues (AA genotype) (Fig. 3e, f). MiR-208a-5p levels were measured in the above cancer tissues and shown non-significant differences between the wildtype and mutant genotype groups (Fig. 3g). Taken together, these results demonstrated that rs79036859 in *DAAM1* 3′-UTR decreases the binding affinity with miR-208a-5p and eliminates miR-208a-5p-mediated decrease of *DAAM1* expression in mammalian cells.

### MiR-208a-5p suppresses the migration of BrCa cells

**Fig. 4:**
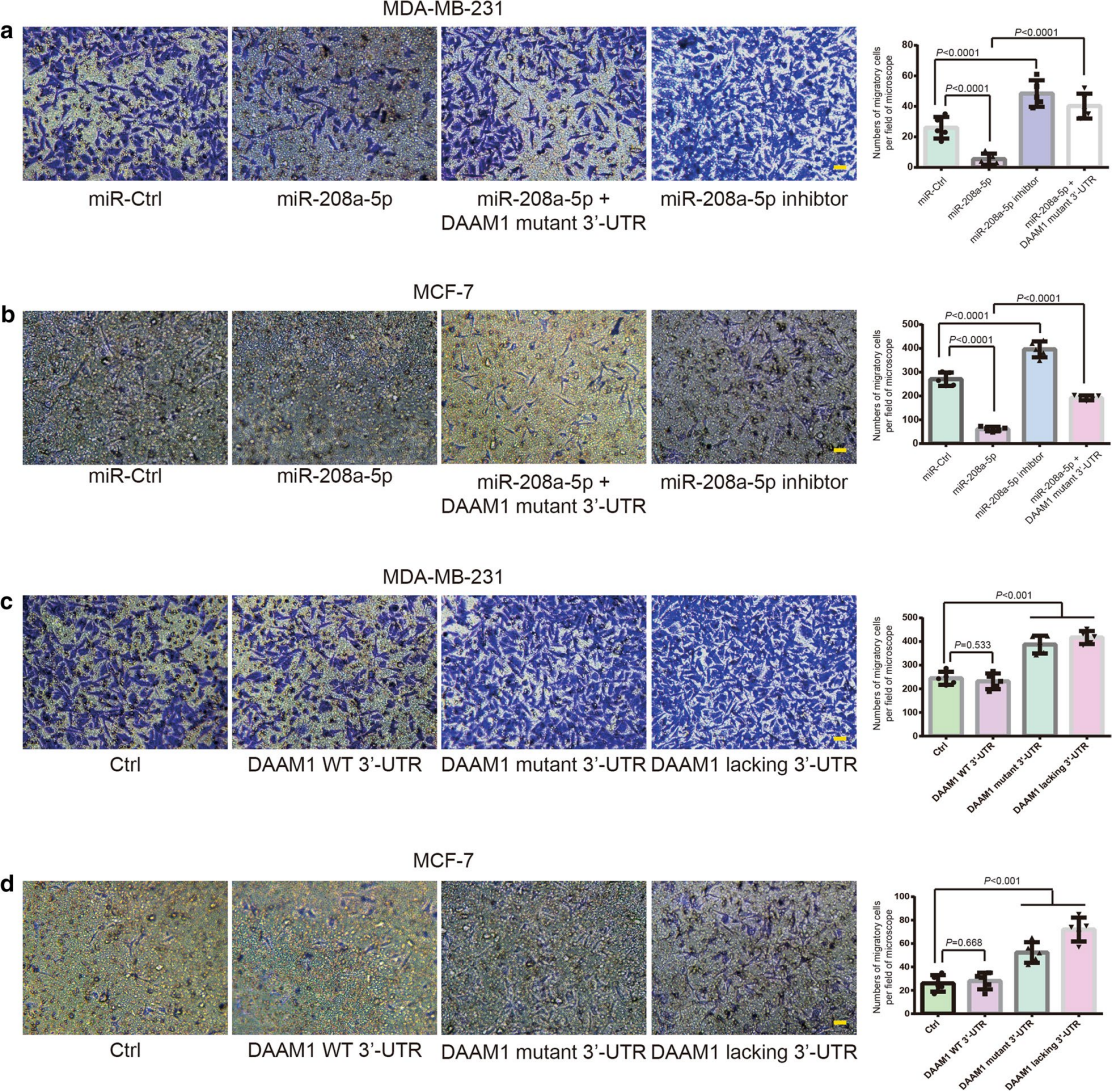
MiR-208a-5p inhibits the migratory ability of BrCa cells. **a**, **b** miR-208a-5p suppressed the migration of MDA-MB-231 and MCF-7 cells, which could be rescued by the overexpression of miR-208a-5p inhibitor (anti-sense of miR-208a-5p) or DAAM1 linking with the G genotype of *DAAM1* 3′-UTR rs79036859 (DAAM1 mutant 3′-UTR). Migratory cells on the lower-side of Boyden chambers were counted per field of microscope. Bar = 20 μm. Objective lens, magnification, × 20; numerical aperture, 0.75. **c**, **d** The overexpression of DAAM1 linking with the G genotype of *DAAM1* 3′-UTR rs79036859 (DAAM1 mutant 3′-UTR) and DAAM1 lacking 3′-UTR elevated the migration of MDA-MB-231 and MCF-7 cells. DAAM1 linking with wildtype 3′-UTR (DAAM1 WT 3′-UTR) failed to promote the migratory rate of MDA-MB-231 and MCF-7 cells. MDA-MB-231 and MCF-7 cells transfected with DAAM1 WT 3′-UTR, DAAM1 mutant 3′-UTR, or DAAM1 lacking 3′-UTR were examined the migration for 8 h in 8.0-µm porous Boyden chamber membranes

Next, we evaluated the role of miR-208a-5p on the migration of BrCa cells which carry wildtype DAAM1 3′-UTR. Boyden chamber assays revealed that miR-208a-5p overexpression suppressed the migratory ability of MDA-MB-231/MCF-7 cells (Fig. 4a, b). On the contrary, impediment of miR-208a-5p binding *DAAM1* 3′-UTR by the overexpression of miR-208a-5p inhibitor (anti-sense of miR-208a-5p) or DAAM1 linking with the G genotype of *DAAM1* 3′-UTR rs79036859 (DAAM1 mutant 3′-UTR) promoted the migration of BrCa cells (Fig. 4a, b). Besides, the overexpression of DAAM1 linking with the G genotype of *DAAM1* 3′-UTR rs79036859 and *DAAM1* lacking 3′-UTR promoted the migration of MDA-MB-231/MCF-7 cells (Fig. 4c, d). DAAM1 linking with wildtype 3′-UTR failed to enhance the migratory rate of BrCa cells (Fig. 4c, d). Thus, these results suggested that miR-208a-5p suppresses cell migration via downregulating DAAM1 in BrCa cells.

### MiR-208a-5p inhibits the RhoA activity and disrupts the formation of microfilament in BrCa cells

**Fig. 5:**
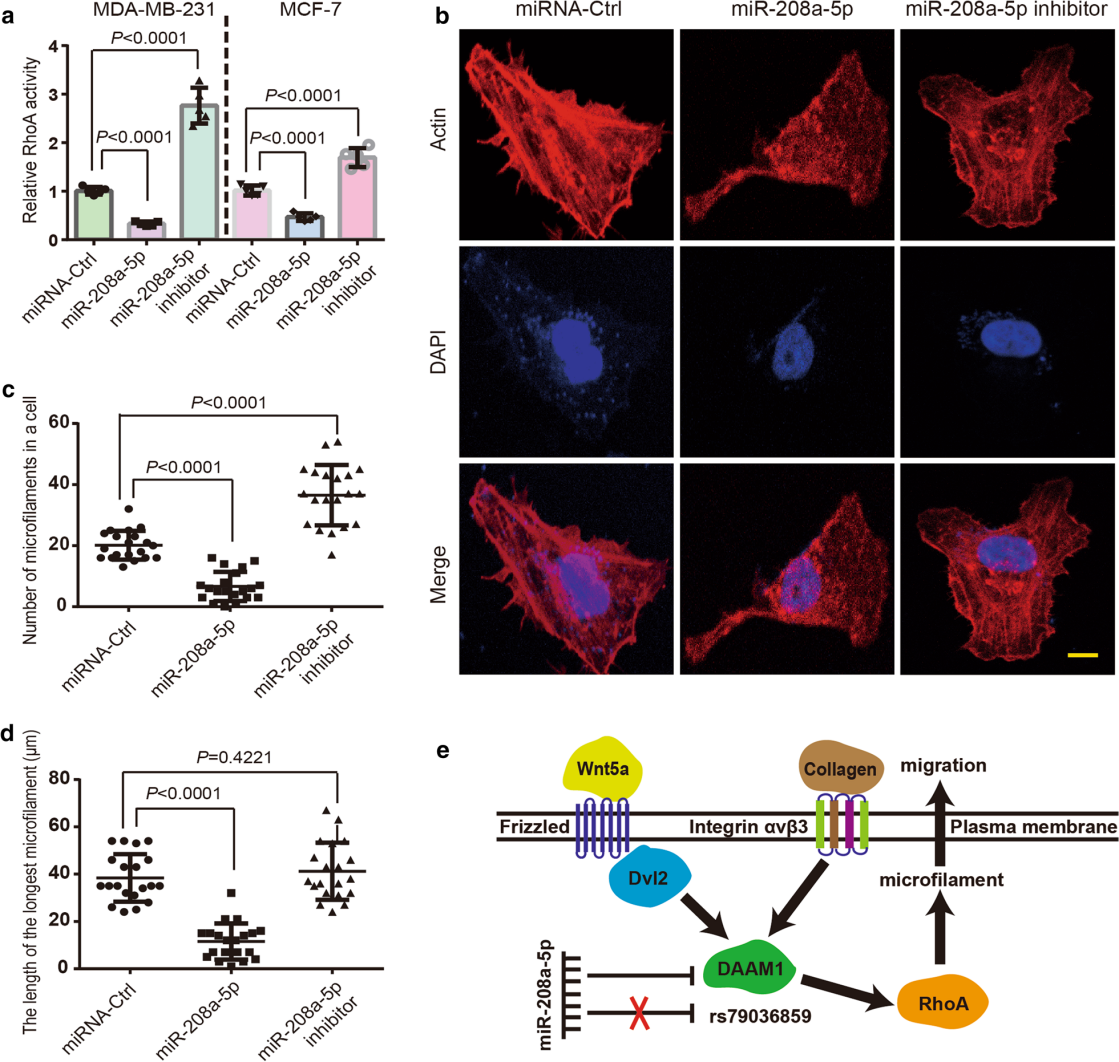
MiR-208a-5p inhibits the RhoA activity and disrupts the formation of microfilament in BrCa cells. **a** RhoA GTPase activation assays showing the downregulated RhoA activity in MDA-MB-231 and MCF-7 cells after miR-208a-5p overexpression. MiR-208a-5p inhibitor (anti-sense of miR-208a-5p) elevated the RhoA activity in MDA-MB-231 and MCF-7 cells. **b** miR-208a-5p overexpression disrupted the formation of microfilament in MDA-MB-231 cells. MiR-208a-5p inhibitor (anti-sense of miR-208a-5p) promoted the formation of microfilament. Bar = 10 μm. Objective lens, magnification, ×40; numerical aperture, 0.95. **c**, **d** The number of microfilaments in each MDA-MB-231 cell and the length of the longest microfilament were measured (n = 20). **e** A schematic diagram of DAAM1 (SNP rs79036859) associated signal transduction pathway and miR-208a-5p locating the upstream of DAAM1

DAAM1 is reported to be involved in Wnt5a-induced and collagen-induced signaling pathways [3, 6]. RhoA is a direct downstream target of DAAM1, which functions in actin assemblage [1, 3, 6]. We investigated the fact that whether miR-208a-5p inhibited the activation of RhoA and the inhibition was reversed by the transfection of miR-208a-5p inhibitor (anti-sense of miR-208a-5p). MiR-208a-5p overexpression notably suppressed RhoA activation, that could be reversed by miR-208a-5p inhibitor overexpression (Fig. 5a). Next, fluorescent phalloidin was stained to display the arrangement of microfilaments in BrCa cells. MiR-208a-5p overexpression obviously blocked the microfilament formation, and miR-208a-5p inhibitors largely enhanced the microfilament assemblage in MDA-MB-231 cells (Fig. 5b–d). Thus, the findings illuminated that active RhoA induced formation of microfilament is disrupted by miR-208a-5p through downregulating DAAM1. Collectively, the findings suggested that the formation of microfilament is disrupted by miR-208a-5p overexpression via targeting DAAM1/RhoA signaling in BrCa cells (Fig. 5e).

## Discussion

The first finding in this study is that the high level of DAAM1 expresses in lymphnode metastatic BrCa tissues. These clinicopathological and biochemical characteristics imply DAAM1 is likely to function as a metastatic promoter in BrCa, which is consistent with our previous findings verified on the cellular levels [3, 6, 17].

Protein is translated from RNA, which is strictly conformed to the genetic central dogma [19]. The mRNA modification exists extensively in eukaryotic cells and leads to the diversity of protein expression. Zhang et al. has analyzed the big data from the Cancer Genome Atlas (TCGA) and identified novel mutations at eQTL situated in *DAAM1* promotor (− 91 bp), which are able to increase *DAAM1* mRNA expression levels [7]. In this study, we focus on the polymorphism of 3′-UTR of *DAAM1* gene. Patients with the G (GG/GA) allele genotypes have the prominently higher risk of metastasis in BrCa than AA genotype of SNP rs79036859. These results demonstrate that G genotype of rs79036859 in *DAAM1* 3′-UTR enhances the risk of metastasis in BrCa.

The most common post-transcriptional regulation is that miRNAs bind to 3′-UTR of target gene and induce the degradation of whole mRNAs. We find that miR-208a-5p targets to the 3′-UTR of *DAAM1* gene. Wang et al. reported the downregulation of miR-208a/miR-208b significantly suppresses the harmful effect of extracellular vesicles on hypoxia/reoxygenation damage in cardiac myoblasts [20]. The regulatory feedback loop of miR-208a-SOX2/β-catenin-LIN28-let-7a-DICER1 mediates the renovation of BrCa stem cells [21]. MiR-208a is capable of promoting gastric cancer progression by suppressing SFRP1 and downregulating MEG3 [22]. Expression analysis demonstrate that miR-208a-5p is significantly suppressed in lymphnode metastatic tumor samples than that in non-lymphnode metastatic tumor samples, indicating the potential role of miR-208a-5p on anti-tumor metastasis via DAAM1 signaling.

DAAM1, an element of cellular actin cytoskeleton, transduces Wnt/Dishevelled signaling to RhoA and then assemble microfilaments in mammalian cells [2, 3, 23]. Actin polymerization is the key step of the assemblage of microfilament in the process of cancer metastasis [24]. The functional and dynamic microfilaments are generally provided a possible route for the degradation of extracellular matrix and available for cancer cells escaping from the primary lesion [24, 25]. Our previous studies find that active DAAM1 is elevated by the treatment of Wnt5a or type IV collagen and participates in the tumor cell migration and haptotaxis [3, 6]. Here, we demonstrate that miR-208a-5p overexpression decreases DAAM1 mRNA and protein expression levels, resulting in the decrease of the migratory ability of BrCa cells. We also find that miR-208a-5p downregulates the RhoA activity, disrupts the formation of microfilaments of BrCa cells. We summarize the potential DAAM1 signaling pathways in Fig. 5e. Thus, miR-208a-5p/DAAM1/RhoA axis is the potential therapeutic target in regulating microfilament assemblage and cell migration.

## Conclusions

These results indicate that SNP rs79036859 G variant of DAAM1 3′-UTR contributes to the likelihood of BrCa metastasis via miR-208a-5p binding capacity. Cell migration is inhibited by miR-208a-5p overexpression via targeting DAAM1/RhoA signaling in BrCa cells. Overall these data identify miR-208a-5p/DAAM1/RhoA axis as the novel therapeutic target in limiting BrCa metastasis.

## Additional file

**Additional file 1.** Associations between miR-208a-5p expression and clinicopathological characteristics in breast cancer.

## Abbreviations

DAAM1: dishevelled-associated activator of morphogenesis 1
BrCa: breast cancer
SNP: single nucleotide polymorphism
UTR: untranslated region
eQTL: expression quantitative trait locus
miRNA: microRNA
IHC: immunohistochemistry
IRS: immunoreactive score
DFS: disease free survival
OS: overall survival
TCGA: The Cancer Genome Atlas
