# Supplementary material for: A DAAM1 3′-UTR SNP mutation regulates breast cancer metastasis through affecting miR-208a-5p-DAAM1-RhoA axis
Source: Cancer Cell Int. 2019 Mar 11;19:55. doi: 10.1186/s12935-019-0747-8 (PMC6417246; doi:10.1186/s12935-019-0747-8)
Supplement: Supplementary file 1 — Additional file 1. Associations between miR-208a-5p expression and clinicopathological characteristics in breast cancer. [file 12935_2019_747_MOESM1_ESM.docx]

**[Additional file 1: Table S1](https://doi.org/10.1186/s12935-018-0681-1)**

**Associations between miR-208a-5p expression and clinicopathological characteristics in breast cancer.**

| **Characteristics** | **n** | **miR-208a-5p ^a^** | | **OR (95%Cl)** | | ***P* value ^b^** |
| --- | --- | --- | --- | --- | --- | --- |
|  |  | **low** | **high** |  |  |  |
| **Tumor size** | | | | | | |
| ≤2cm | 61 | 29 | 32 | | 0.89 (0.46-1.71) | 0.716 |
| >2cm | 87 | 44 | 43 | |  |  |
| unknown | 9 |  |  | |  |  |
| **Lymph node metastasis** | | | | | | |
| N0 | 121 | 59 | 62 | | 0.85 (0.40-1.79) | 0.672 |
| N1-N3 | 36 | 19 | 17 | |  |  |
| **Distant metastasis** | | | | | | |
| M0 | 151 | 73 | 78 | | 0.19 (0.02-1.64) | 0.093 |
| M1 | 6 | 5 | 1 | |  |  |
| **ER status** | | | | | | |
| negative | 51 | 23 | 28 | | 0.76 (0.39-1.49) | 0.426 |
| positive | 106 | 55 | 51 | |  |  |
| **PR status** | | | | | | |
| negative | 67 | 32 | 35 | | 0.89 (0.47-1.69) | 0.729 |
| positive | 89 | 45 | 44 | |  |  |
| unknown | 1 |  |  | |  |  |
| **Her-2 status** | | | | | | |
| negative | 94 | 50 | 44 | | 1.43 (0.75-2.74) | 0.277 |
| positive | 61 | 27 | 34 | |  |  |
| unknown | 2 |  |  | |  |  |
| **P53 status** | | | | | | |
| negative | 105 | 57 | 48 | | 1.86 (0.89-3.87) | 0.097 |
| positive | 41 | 16 | 25 | |  |  |
| unknown | 11 |  |  | |  |  |
| **Ki67 status** | | | | | | |
| negative | 33 | 13 | 20 | | 0.60 (0.27-1.31) | 0.196 |
| positive | 119 | 62 | 57 | |  |  |
| unknown | 5 |  |  | |  |  |

^a^ The miR-208a-5p expression levels were divided at a cutoff point of 50%.

^b^ *P* value for χ^2^ test.
